# Digital Health for Medication Adherence in Adult Diabetes or Hypertension: An Integrative Review

**DOI:** 10.2196/diabetes.8030

**Published:** 2017-08-16

**Authors:** Cheryl Moseley Conway, Teresa J Kelechi

**Affiliations:** 1 School of Nursing Western Carolina University Cullowhee, NC United States; 2 College of Nursing Medical University of South Carolina Charleston, SC United States

**Keywords:** digital health, medication adherence, Chronic Care Model, diabetes, hypertension

## Abstract

**Background:**

Optimal management of chronic diseases, such as type 2 diabetes and hypertension, often include prescription medications. Medication adherence (MA) is one component of self-management. Optimization through digital health—eHealth and mHealth—could enhance patient awareness and/or communication between the patient and provider.

**Objective:**

Medication adherence is a major issue that affects 50%-60% of chronically ill adults. Digital health refers to eHealth and mHealth, collectively, and as these technologies become more accessible, remote health delivery is increasingly available as an adjunct to improve medication adherence; communicate with patients and providers; and provide education to patients, families, and communities. The objective of this integrative review was to examine the types of digital health technologies that targeted medication adherence in the adult population with diabetes or hypertension.

**Methods:**

An integrative review was conducted using databases within EBSCOhost, PubMed, and Scopus. Eligible studies available as of September 2016 had to be written in English, had to contain digital health interventions to improve medication adherence to prescription medications in adults 18 years or older, and had to focus on diabetes or hypertension.

**Results:**

Of the 337 located studies, 13 (3.9%) used a digital health intervention for medication adherence to prescribed medications for diabetes or hypertension and were assessed according to the Chronic Care Model.

**Conclusions:**

The 13 studies included in this review found no conclusive evidence of improved medication adherence using digital health interventions such as interactive voice response (IVR), short message service (SMS) text messaging, telemonitoring, and interactive software technology. Among the 13 studies were digital health interventions that foster medication adherence via one-way communication to the patient or two-way communication between the patient and health care provider for adjunct medication adherence strategies. More research is needed to determine which digital health interventions are most beneficial for individuals with diabetes or hypertension.

## Introduction

### Background

The effect of chronic diseases on the health and wellness of individuals is increasing in every region of the world. The World Health Statistics 2012 report states that one in three adults worldwide has raised blood pressure and one in 10 adults has diabetes [1]. Further, diagnosis and treatment with low-cost medication has reduced mean blood pressure across populations in high-income countries and has the potential to reduce death and disability in low-income countries [1]. Globally, there are approximately 422 million adults with diabetes as compared to 108 million in 1980 (4.7%-8.5% in the adult population) [2]. Chronic conditions such as diabetes and hypertension contribute to an international chronic disease burden and negatively influence patient health outcomes [1,2].

Treatment of chronic conditions often includes prescription medications. Nonadherence to medication therapy can compound the increases in morbidity and mortality, and can further add to additional health care costs [3]. It is estimated that increased prescription medication adherence (MA) could save the United States US $5 billion annually in health care costs, including decreasing expensive emergency department visits and hospitalizations [4]. Medication nonadherence contributes significantly to the growing burden of disease and high costs associated with care [3,5,6].

The purpose of this integrative review is to assess the benefits of using digital health technology to improve medication adherence for diabetes and hypertension in the adult population. The following questions were asked to guide the review:

1. Does digital health technology improve medication adherence in adults with diabetes or hypertension?
2. What are benefits and barriers of medication adherence technology when implemented in adults with diabetes or hypertension?

### Medication Nonadherence is a Major Problem

Medication nonadherence for individuals with chronic diseases such as diabetes and hypertension has been established as a major factor associated with negative patient outcomes [3,7]. Medication nonadherence is defined as taking less than 80% of prescribed doses, without exceeding recommended dosing [8,9]. Medication nonadherence is a complex issue with many contributing factors, which have been categorized as individual related (ie, forgetfulness and low health literacy skills) and medication related (ie, increasing dosage, increased number of medications, poor communication skills of provider, and lack of medication review by provider) [8]. The landmark World Health Organization report from 2003 stated that nonadherence to taking medications in developed countries is 50% [10], underscoring that nonadherence to prescription medication remains a significant issue, not only in the United States, but worldwide.

### Measuring Medication Adherence/Nonadherence Remains Problematic

Methods to measure medication adherence fall into three categories: subjective, objective, or biochemical marker analyses. Subjective measurement is obtained by asking the patient, family member, caregiver, or physician about medication use [9]. Objective measurement is obtained by pill count, pharmacy refill information, electronic pharmacy refill data, serum drug level, or levels in the blood or urine [3,9,11]. Additionally, the use of biomedical measures is objective and accurate; however, they are expensive [11]. Technology-based methods have been introduced that provide digital health options. Whether these digital health devices improve adherence remains a topic of debate in the literature.

### Health Technology to Promote Medication Adherence

Telehealth is the use of electronic information and telecommunication technologies to support clinical health care from a distance, patient and professional health education, as well as public health and health administration [12]. Additionally, telehealth includes preventive and curative health care delivered over a distance, and all forms—electronic health (eHealth), telehealth, and telemedicine—are intersected by mobile health (mHealth) [13]. A number of electronic (ie, eHealth), mobile (ie, mHealth), telehealth, and telemedicine methods have been developed to improve the delivery of health care for various conditions. eHealth refers to secure cost-effective use of information and communication technologies specific to health and health-related fields [14]. mHealth is a component of eHealth and includes mobile technologies used for dissemination of health services and information (ie, mobile phones, monitoring devices, tablets, personal digital assistants, and wireless devices) [14-16]. mHealth promotes the individual’s interaction with an electronic device or technology to access or receive health information, directions, or support about health [17]. Digital health refers to eHealth and mHealth collectively [14].

In addition to facilitating communication among health care providers, these modalities can provide the opportunity for patients to receive one-way communication about health conditions and two-way communication with providers that is tailored to a health condition; this includes health data that can be transmitted as well as collected [15,16,18,19]. The diverse nature of digital health modalities as well as the evolving nature of technology provide both an opportunity and challenge for health care providers who seek to integrate technology into patient care. A plethora of data exists on the use of digital technology to assist with medication adherence [20-23]; however, further exploration is needed to determine whether these modalities improve and subsequently enhance chronic disease self-management, in particular, medication adherence in adults.

Findings from studies including digital health devices have shown improvements in self-management and adherence to treatments in many conditions such as asthma, chronic obstructive pulmonary disease, hypertension, and diabetes [24-26]. A Cochrane review of mobile phone messaging for self-management of chronic disease reported medication compliance in hypertension was 8.9% higher in the short message service (SMS) text message group versus the control group [27]. Several studies have indicated that interactive voice response (IVR) and SMS text messaging foster medication adherence through telephone-delivered diabetes education and interactive reminders can improve medication adherence in patients with diabetes [20,21,23]. Mobile communication also includes one-way and two-way text messages and weekly IVR calls to promote medication adherence for low-income racially and ethnically diverse adults with type 2 diabetes [28,29]. Additionally, telemonitoring, telehealth, and the use of a virtual classroom have been shown to enable the individual with diabetes to participate in adherence strategies [22,30]. These digital health strategies provide interactive communication that is timely and patient centered. The tailored information provided has the potential to improve patient outcomes through education and timely information.

**Table 1:** Chronic Care Model^a^ components and descriptions.

Chronic Care Model components	Descriptions
Self-management support	Designed to inform the patient and family by providing training and promotion to foster self-management. The eCCM^b,c^ further adds 24/7 access, convenience, reminders, and alerts.
Decision support	Emphasizes the goal to improve medical decisions for providers and patients to access current evidence-based care guidelines, reminders, and information buttons.
Clinical information systems	Collects, maintains, and utilizes patient registries; develops patient portals, Internet, mHealth, mobile phones, wearable devices, electronic health records, and personal health records.
Delivery system design	Emphasizes the importance of interdisciplinary clinical teams and collaboration between the patient and multiple providers.
Community support	Health care organization makes an effort to form powerful alliances and partnerships. The eCCM^c^ further adds that eHealth education is included as a component, including eCommunity and encompassing message training, health education, technology training, numeracy, literacy, usability, and security.
Health systems	Creates an environment in which organizational efforts improve health care.

^a^The Chronic Care Model includes self-management support, decision support, clinical information systems, delivery system design, community support, and health systems as interdependent components for holistic care.

^b^eCCM: eHealth-enhanced Chronic Care Model.

^c^The eCCM further defines the role of eHealth tools and eCommunity to support holistic care.

### Theoretical Framework

The Chronic Care Model (CCM) (see Table 1) is a well-established, validated framework to provide a caring approach for chronically ill individuals with a focus on increasing function and improving clinical outcomes [31].

The CCM postulates that optimal care for individuals with chronic illness requires a health system that provides the following: community support, self-management support, decision support, clinical information systems, and delivery system design [32]. Further, the eHealth-enhanced CCM (eCCM) includes the role of eHealth tools in self-management for individuals with chronic illness [31]. The eCCM is particularly tailored for assessing digital health findings as compared to the CCM, due to the inclusion of eHealth tools and strategies. This is also due to the broader definition of eCommunity to encompass a broader definition of digital health support available to include community support and education. The CCM and the eCCM are interdependent with the eCCM further defining the significance of eHealth [31].

CCM-based interventions were effective in improving clinical, behavioral, psychological/psychosocial, and diabetic knowledge outcomes, including medication adherence in patients with diabetes in research that did not utilize digital health interventions [33]. The CCM has been used as a framework for care in Malaysia and was found to improve patient outcomes [32] as well as practice-based care delivery redesign [33,34].

With developments in digital health, technology has further incorporated medication adherence strategies for chronic illnesses such as diabetes and hypertension [33-36]. These factors are interrelated, and the CCM has been implemented across many chronic conditions such as asthma, bipolar disorder, breast cancer, diabetes, hypertension, and obesity [37]. For the purpose of this integrative review, the CCM will be used to evaluate the use of digital health technology for medication adherence in diabetes or hypertension.

## Methods

**Figure 1:**
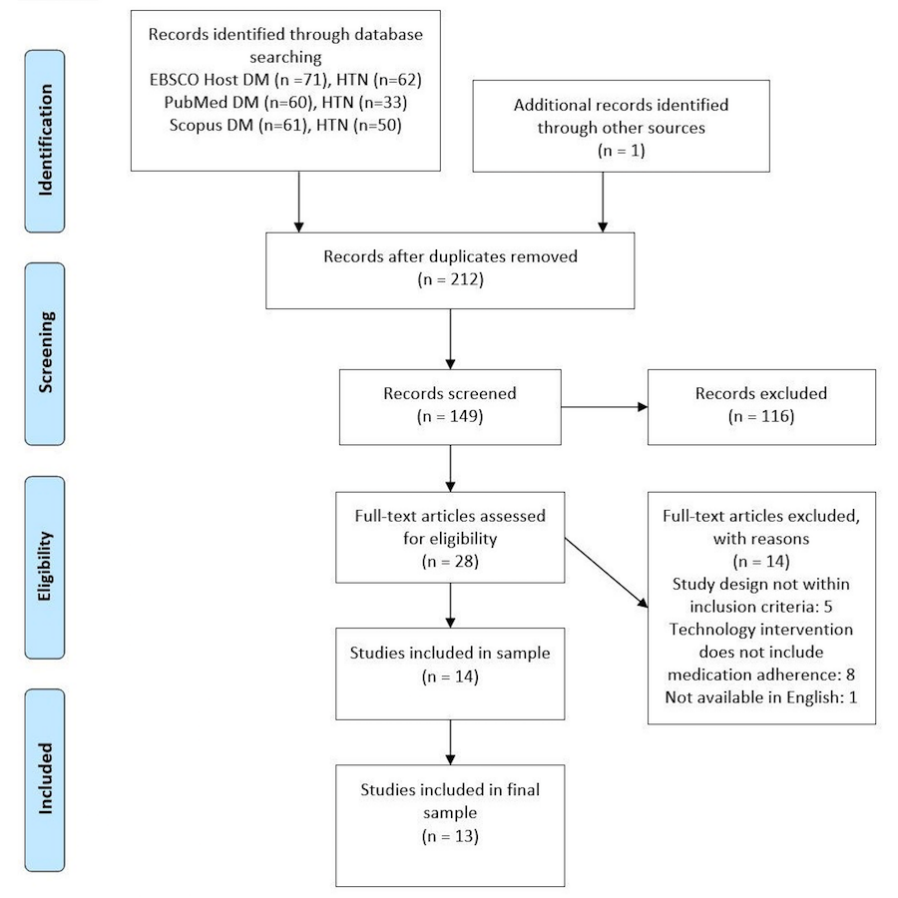
Preferred Reporting Items for Systematic Reviews and Meta-Analyses (PRISMA) flowchart of studies from search to inclusion [39]. DM: diabetes; HTN: hypertension.

This integrative review adhered to the following five stages: (1) problem identification, (2) literature search, (3) data evaluation, (3) data analysis, and (5) presentation [38]. The Preferred Reporting Items for Systematic Reviews and Meta-Analyses (PRISMA) flowchart in Figure 1 [39] was used to depict the search results. Using a two-step strategy, a literature search was conducted to find relevant studies published from January 2006 to October 2016. Consultation with a health reference librarian aided in the refinement of search terms in the databases.

In the first step, a search strategy was developed. One reviewer (CMC) conducted the search using the following databases included within EBSCOhost to ascertain relevant studies: PubMed and Scopus. A combination of keywords and Medical Subject Headings (MeSH) terms were used as follows: “mobile health,” “mHealth,” “telemedicine,” “eHealth,” “remote consultation,” *or* “digital health” *and* “medication adherence,” “medication nonadherence,” “medication compliance,” “medication noncompliance,” *or* “medication persistence” *and* “diabetes mellitus,” “diabetes,” “type 2 diabetes,” *or* “type 2 diabetes mellitus” *and* “technology,” “websites,” *or* “apps.” Reference lists of relevant studies were hand searched. Identified citations were exported to Endnote reference management program. This strategy initially yielded 337 studies.

For inclusion in this review, peer-reviewed studies were required to report on a digital health intervention for medication adherence. Inclusion criteria included (1) English-language, peer-reviewed randomized controlled trials (RCTs) with quasi-experimental, observational, or qualitative design; (2) studies containing digital health interventions to improve medication adherence to prescription medications in adults (ie, 18 years or older); and (3) studies focused on diabetes or hypertension. Exclusion criteria included (1) studies that did not include results of medication adherence rates or (2) pilot studies. Titles and abstracts were reviewed for relevance.

## Results

### Overview

Of the 337 studies, 13 (3.9%) [22,26,40-50] used a digital health intervention to promote medication adherence to prescribed medications for diabetes and hypertension that was summarized (see Multimedia Appendix 1) and evaluated with the Chronic Care Model (see Table 2).

Studies included nine RCTs, one quasi-experimental study, and two observational studies, one of which was a mixed-methods design. Most studies were conducted in the United States, with one study each conducted in the United Kingdom, South Africa, and South Korea.

A total of 13 studies were selected, analyzed, and organized (see Multimedia Appendix 1). Medication adherence findings for the intervention and data extraction categories, including the study objective, design, sample, intervention length, and participant age, are included in Multimedia Appendix 1. Strategies used to improve medication adherence included four primary approaches: IVR (with or without human interaction), SMS text messaging, telemonitoring and/or tailored care management, and Web-based software. The subheadings in this section consist of progressively interdependent components of the Chronic Care Model that influence patient-centered, evidence-based care and are designed to improve health outcomes by changing the routine delivery of care (ie, self-management support, decision support, clinical information systems, delivery system design, community support, and health systems) [37]. The reviewed studies are presented in Multimedia Appendix 1 with the intent to categorize findings based on CCM components in order to assess findings about digital health interventions for medication adherence. Table 2 provides a summary of CCM components used in each study [22,26,40-50].

**Table 2:** Chronic Care Model applied to studies.

Study author, year	Chronic Care Model components used in studies^a^				
	Self-management support	Decision support	Clinical information systems	Delivery system design	Community support
Aikens et al, 2014 [40]	X			X	X
Arora et al, 2014 [41]	X				
Bobrow et al, 2016 [42]	X				
Davidson et al, 2015 [43]	X	X	X		X
Edelman et al, 2015 [44]	X	X		X	X
Katalenich et al, 2015 [45]	X	X		X	
Kim et al, 2006 [46]	X		X		
Migneault et al, 2012 [48]	X	X			
Nelson et al, 2016 [47]	X				
Nundy et al, 2014 [49]	X				X
Shane-McWhorter et al, 2014 [22]			X	X	
Wakefield et al, 2011 [26]				X	
Wild et al, 2016 [50]			X	X	

^a^The health systems component was not found in the included studies.

### Application of Chronic Care Model in Aim 1

#### Self-Management Support

The goal of *self-management support* is to inform the patient and family by providing training and health promotion to foster self-management [37]. The eCCM further adds 24/7 access, convenience, reminders, and alerts [31]. In a study conducted by Aikens and colleagues [40], a combined program of automated telemonitoring, clinician notification, and informal caregiver involvement was found to be associated with improvements in medication adherence between pretest (mean 1.20, SD 0.95) and posttest (mean 0.87, SD 0.88) using linear regression analyses. This observational, open-label trial of 301 adults aimed to identify changes in diabetes self-management and psychological distress associated with an mHealth, IVR, self-management support program [40]. Arora and colleagues [41] conducted an RCT of 128 adult patients with poorly controlled diabetes in an urban emergency department with a unidirectional, SMS text message-based mHealth intervention in English or Spanish; the RCT was called the Trial to Examine Text-based mHealth for Emergency department patients with Diabetes (TExT-MED). There was improved medication adherence among the TExT-MED group compared to the control group as measured by the Morisky Medication Adherence Scale (MMAS) (score of 4.5 to 5.4) [20]. Bobrow and colleagues [42] found that interactive SMS text messaging subsequently improved medication adherence in a group of 1372 patients treated for hypertension as indicated by prescription refill data (ie, had at least 80% of the days covered). Refill rates were higher among the information-only message group (156/248, 62.9%) and interactive message group (134/225, 59.6%) compared to the usual care group (94/190, 49.5%) [42]. Similarly, Davidson and colleagues [43] found a cellular-connected, electronic medication device that provided reminder signals and mobile phone messaging; 38 patients were reminded to take their blood pressure (BP) medications using a Bluetooth-accessible, BP monitor-improved medication adherence device in African American and Hispanic participants. Medication adherence was defined by the percent of SMS text message reminders over the past day(s) and the mean medication adherence score was 92 (SD 0.09) for all participants in the intervention group [43]. In an RCT of patients with diabetes and hypertension, Edelman and colleagues [44] found that 377 participants receiving tailored, focused behavioral content had improved medication adherence obtained by self-report. The secondary outcome of nonadherence was 52 out of 193 (26.9%) for the diabetes intervention group and 58 out of 184 (31.5%) for the control group as reported by the medication-taking scale [44]. Similarly, Katalenich and colleagues [45] found that engagement in the automated Diabetes Remote Monitoring and Management System improved medication adherence as measured by the MMAS, although the improvement was not statistically significant. The intervention group (50/98, 51%) had higher adherence (28%, 26%, and 27%) than the control group (48/98, 49%) (12%, 22%, and 20%) at baseline, 3 months, and 6 months, respectively; however, overall improvements in medication adherence self-report were not significant [45]. A quasi-experimental study conducted by Kim and colleagues [46] found that SMS text messaging from nurses by mobile phone or the Internet improved medication adherence in 45 patients with diabetes. Self-reported medication adherence was measured by the Summary of Diabetes Self-Care Activities (SDSCA) measure; diabetes medication-taking adherence increased 1.1 days per week at posttest as compared to pretest [46]. Recently, Nelson and colleagues [47] leveraged IVR and SMS text messaging in 80 patients with diabetes using MEssaging for Diabetes (MED) and found short-term improvements in medication adherence among adults with type 2 diabetes. Medication adherence was assessed using the SDSCA medications subscale and improved in the intervention group at 1 month (mean 6.5, SD 1.4) and 2 months (mean 6.8, SD 0.4), but did not continue to improve at 3 months (mean 6.2, SD 1.3) [47]. Likewise, an RCT conducted by Migneault and colleagues [48] found that a culturally adapted, automated telephone system used among 337 hypertensive, urban African American adults improved medication adherence slightly as measured using the MMAS (0.19 points relative to controls), which was not statistically significant. Finally, Nundy and colleagues [49] conducted a mixed-methods observational cohort study using a theory-driven, mobile phone-based intervention with 74 adults with diabetes; an automated SMS text messaging system combined with remote nursing improved medication adherence as measured by the SDSCA measure of weekly adherence and the MMAS. At both 3 and 6 months, the MMAS 4-item score (out of 4) improved (3.3, *P*<.10 and 3.4, *P*<.02, respectively) compared to baseline (2.9, *P*<.10); however, no change in weekly medication adherence was observed between 3 and 6 months (score of 4.4 and 4.4, respectively) [49]. There are positive benefits of the use of one-way and two-way digital health messages to engage patients in timely self-management to improve medication adherence. Strategies such as IVR and SMS text messaging provide evidence of improved short-term medication adherence using educational and reinforcement reminders.

#### Decision Support

*Decision support* emphasizes the goal to improve medical decisions for providers and patients to access current evidence-based care guidelines [37]. In addition, eCCM discusses reminders and info buttons [31]. Davidson and colleagues [43] discussed a “several year” iterative design process for the Smartphone Medication Adherence Stops Hypertension (SMASH) program that involved key informant interviews and focus groups with health care providers and patients to develop SMS text message reminders. Likewise, Edelman and colleagues [44] used nurses’ behavior-modifying content specific to each patient’s individual barriers based on evidence-based approved content. In addition, Migneault and colleagues [48] developed the Telephone-Linked-Care intervention using ethnic mapping in focus groups for hypertension. In addition, Katalenich and colleagues [45] used validated diabetes algorithms to evaluate glycemic control and adherence. Of the four studies that included decision support for medication adherence, two had statistically significant findings [43,44] while two did not have statistically significant differences between groups [45,48].

#### Clinical Information Systems

*Clinical information systems* are used to collect, maintain, and utilize information within the context of health care, such as patient registries and electronic medical records [31]. In addition, eCCM emphasizes the development of patient portals, Internet, mHealth, mobile phones, wearable devices, and patient health records [31]. Integration of secure-messaging e-visits, home monitoring with feedback, health risk appraisal with feedback, medication refills, tailored interventions, and links to community programs are possible with digital health technology. In a study by Davidson and colleagues [43] to measure medication adherence, electronic medication trays provided reminder signals and SMS text messaging reminded 38 African American and Hispanic participants to monitor BP with Bluetooth-enabled monitors; a mean of 92% (SD 0.09) of reminders were received across the 6-month trial. Additionally, an MA (medication adherence) score was averaged to calculate adherence (daily scores ranged from 0 to 1) with “fully compliant” defined as ingesting all medications within a 3-hour window. Ingesting all medications within a 6-hour window received half credit and ingesting all medications outside a 6-hour window or a missed dose received no credit. The mean MA score was 92 (SD 0.09) for all participants [43]. Additionally, the intervention group BP mean adherence was 86.2% (SD 6) (on time every 3 days) [43]. Recently, Katalenich and colleagues [45] studied health care providers who could monitor progress of their 98 patients through a Web-based secure portal; study findings revealed the intervention group (50/98, 51%) had higher MA than the control group (48/98, 49%) at each measurement time—baseline, 3 months, and 6 months. Additionally, Kim and colleagues [46] included the use of the Internet to support secure communication-based optimal diabetes recommendations, with the intervention group (33/45 completers, 73%) having an increased MA of 1.1 days per week between pretest and posttest. A study by Shane-McWhorter and colleagues [22] discussed the asynchronous involvement of a remote care monitor—usually a pharmacist—and email alerts to a medical provider if a patient has an out-of-range value via a mobile communication platform. The nonrandomized prospective observational preintervention and postintervention design of the study with 125 participants resulted in improvements in medication adherence for diabetes patients (6.2 and 6.5, respectively; *P*=.09) and hypertension patients (6.3 and 6.7, respectively; *P*=.05); however, the difference in improvement was not statistically significant for the intervention group. An RCT conducted by Wild and colleagues [50] that included 321 participants with type 2 diabetes used Bluetooth technology to transmit BP, glucose, and weight readings through a supplied modem interacting with a remote secure server manned by research nurses. Medication adherencewas reported with no significant differences between the monitored intervention group (n=160) and control group receiving usual care (n=161). MA linear regression models were performed for 270 participants: monitored group (n=139; baseline and follow-up mean 0.7, SD 0.9) and unmonitored/control group (n=131; baseline mean 1.0, SD 1.0; follow-up mean 0.8, SD 1.0). Of the four studies that included medication adherence clinical information support using digital health technology, there were no statistically significant findings; supportive measures, such as secure portal, Internet, or medication pill dispenser, were not discussed in the outcomes.

#### Delivery System Design

*Delivery system design* includes the importance of interdisciplinary clinical teams and collaboration between the patient and multiple providers [37]. Bluetooth-enabled devices and the use of chat, voice, and video communication allow the health care team to provide many of the elements of a traditional office visit. The use of innovative technology affords a low-cost, flexible means to supplement formal health care. Aikens and colleagues [40] found that automated telemonitoring clinician notification provided the clinician with actionable feedback through faxed updates about patient-reported health and self-care problems including MA. Aikens and colleagues [40] identified significant pre-post improvement in MA (mean MMAS score 1.20, SD 0.95 and mean MMAS score 0.87, SD 0.88, respectively; *P*<.001). Additionally, Edelman and colleagues [44] reported that a nurse-led diabetes and hypertension behavior modification intervention, Tailored Case Management for Diabetes and Hypertension (TEACH-DM), communicated patient results to providers with statistically insignificant differences between groups as follows: 26.9% from the diabetes intervention group and 31.5% from the diabetes control group were nonadherent (medication-taking scale); 43.0% from the hypertension intervention group and 42.9% from the hypertension control group were nonadherent [44]. Similarly, providers in the Diabetes Remote Monitoring and Management System study could monitor the progress of their patients through a Web-based portal [45]. Overall improvements in medication adherence self-report were not statistically significant [45]. Shane-McWhorter and colleagues [22] used remote telemonitoring for patients with uncontrolled diabetes and/or hypertension from four rural and primary clinics and one stroke center with improvement in medication adherence for diabetes and hypertension, although improvements were not statistically significant (*P*=.09 and *P*=.05, respectively). Wakefield and colleagues [26] used a home telemonitoring device and home care management in patients with comorbid diabetes and hypertension. Medication adherence improved over time for all three groups—high intensity, low intensity, and usual care—but there were no differences among the three groups [26]. Lastly, Wild and colleagues [50] included supported telemonitoring intervention involving self-measurement and transmission to a secure website with no significant differences identified between groups in medication adherence. Of the six studies that included MA delivery system design, one reported statistically significant improvement [40]. The conflicting results from these studies suggest that more research is needed to determine which groups might benefit from digital health strategies.

#### Community Support/eHealth Education

*Community support* links the patient to local resources and provides an opportunity for organizational leaders to establish new relationships and expand [37]. In the eCCM model, *eHealth education* is included as a component of eCommunity and encompasses message training, health education, technology training, numeracy, literacy, usability, and security [31]. Two studies in this review included culturally attuned messages to improve medication adherence [43,48]. Additionally, two studies found a positive correlation between social support and medication adherence [40,49]. One study addressed health literacy, but did not find a significant correlation to medication adherence [44].

#### Health Systems

The health care system creates an environment in which organization efforts improve care [37]. No studies included organization of health care and health systems.

### Benefits and Barriers of Medication Adherence by Digital Health Technology in Aim 2

The second aim of this review was to determine the benefits and barriers of MA technology studied in adults with diabetes or hypertension. Overall, the strongest benefit of digital health technologies to measure medication adherence involve patient engagement in diabetes and hypertension self-management through either one-way or two-way interactive reminders or educational information. Some reminders were culturally adapted [43,48] as well as tailored to the population of interest [40,44,47]. In addition, patient-reported data (ie, medication-taking behaviors, blood glucose, blood pressure, and weight) could be shared with health care providers through interactive communication platforms using SMS text messages, Bluetooth-enabled devices, or the Internet.

The primary barriers of digital health technologies for measurement of MA included the iterative nature of tailored message development, which involved input from focus groups, health care providers, and patients [40,43,44,47], as well as staffing the interactive application [22,26,46,50]. While there were varying costs, there were also ongoing expenses of maintaining a communication platform and/or personnel. The included studies discussed the expense of maintaining telemonitoring infrastructure [50], personnel [22,26], Web-based software [47,48], and electronic medication trays [43].

## Discussion

### Improvement of Medication Adherence Using Digital Health Technology

The first aim of this review was to determine if digital health technologies improve medication adherence in adults with diabetes or hypertension. Of the 13 studies included in this review, there was no conclusive demonstration of improved medication adherence using digital health interventions such as IVR, SMS text messaging, telemonitoring and remote monitoring, and interactive software technology. However, in some studies the benefits of digital health technology were short term or close to statistically significant [47]; for example, benefits improved but were not statistically significant [22] or there were benefits in both the intervention and control groups [26].

**Table 3:** Benefits and barriers of digital health technology for medication adherence.

Study author, year	Digital health technology	Benefits	Barriers
Aikens et al, 2014 [40]	IVR^a^-tailored text messages; clinician notification	Interactive, tailored SMS^b^ text messages; clinician notification	Development of tailored SMS text messages
Arora et al, 2014 [41]	Daily SMS text messages in English or Spanish	Frequent reminders, available in English or Spanish	One-way reminders
Bobrow et al, 2016 [42]	SMS text messaging	SMS text message reminders by mobile phone	Use of refill data; availability of medications
Davidson et al, 2015 [43]	Electronic medication trays: reminder signals; reminder SMS text messages	Culturally sensitive	Expense of electronic medication trays
Edelman et al, 2015 [44]	TEACH-DM^c^: call from nurse; tailored SMS text messages; diabetes- and hypertension-focused content versus nontailored, noninteractive information	Tailored SMS text messages	Development of tailored SMS text messages
Katalenich et al, 2015 [45]	SMS text messages or phone call reminders to use automated system; no interaction unless severely high or low glucose	SMS text message reminders by mobile phone	No interaction unless severely high or low glucose
Kim et al, 2006 [46]	SMS text messages and Internet education	Medication reminders and education	Nurse SMS text message expense
Migneault et al, 2012 [48]	Automated, multi-behavior intervention or education-only control	Culturally adapted reminders and education	Expense of computer-based, interactive counseling system; development of tailored SMS text messages
Nelson et al, 2016 [47]	SMS text messages/IVR: deliver and tailor text messages and voice communications to promote MA^d^	Tailored SMS text messages and IVRs	Expense of communication platform; development of tailored SMS text messages
Nundy et al, 2014 [49]	Web-based software reminders and texted-back responses to questions	Interactive (text-back), mobile-based, educational messages and reminders	Expense of Web-based software
Shane-McWhorter et al, 2014 [22]	Telemonitoring with asynchronous measurements transmitted from the patient to a remote care coordinator: pharmacist or certified diabetes educator	Self-management; patient data entered for clinician review	Expense of remote care coordinator
Wakefield et al, 2011 [26]	Closed surveillance via home telehealth device and nurse care management	Self-management; patient data entered for clinician review	Expense of nurse care management
Wild et al, 2016 [50]	Supported telemonitoring intervention involved self-measurement and transmission to a secure website; review by family practice clinicians	Clinician notification	Expense of telemonitoring infrastructure and password-protected server

^a^IVR: interactive voice response.

^b^SMS: short message service.

^c^TEACH-DM: Tailored Case Management for Diabetes and Hypertension.

^d^MA: medication adherence.

While studies demonstrated improved v [40,41], there was no consistent evidence of sustained adherence. Notably, nine studies were 6 months or less in duration. In five studies, there was improvement in medication adherence, but the difference in medication adherence was not statistically significant between the intervention and control groups [22,26,44,48,50].

Different psychometric instruments informed adherence measurement across populations. Medication adherence self-report was the primary method, with MMAS being the most commonly used psychometric instrument, either as the sole measure or in conjunction with another measure such as a self-report questionnaire [40,41,45,48-50]. The MMAS is a well-validated and reliable psychometric instrument to measure medication adherence in populations [51]. Although all 13 studies measured medication adherence, there was a lack of a consistent measure among studies to assess medication adherence, which may partially account for mixed findings of significance in the study interventions.

This review illustrates that digital health interventions hold promise for improving short-term medication adherence for diabetes and hypertension, including IVR, SMS text messaging, telemonitoring, and Web-based software. Nevertheless, despite the growing interest in the use of various digital health technologies, there is limited evidence of efficacy of such interventions for enhancing long-term medication adherence among adults with diabetes or hypertension. Thus, there are still areas in which to learn about medication adherence digital health interventions, such as long-term outcomes, cost-effectiveness, and impact of patient age, ethnicity, and socioeconomic status. Digital health technologies are a promising option. Digital health technologies have improved medication adherence and self-care for some patients with chronic obstructive pulmonary disease [52], coronary artery disease [53], and heart failure [54,55]. More research is needed in adult populations with chronic illnesses and for longer study durations than 6 months using evidence-based, common assessment strategies; these will determine which patients and demographic groups can benefit from digital health interventions for medication adherence. Table 3 provides a summary of the benefits and barriers of the included studies.

### Chronic Care Model

This review included MA interventions categorized according to the Chronic Care Model. In addition, health care providers could use the CCM to provide a blueprint to support care that is evidence based, population based, and patient centered [56] in order to improve digital health intervention-driven outcomes. While the CCM guided this integrative review, other theoretical models could be considered to review digital health technology interventions, such as the Theory of Planned Change or the Technology Acceptance Model. Additionally, the eCCM should be further used and evaluated for validation in chronically ill populations. Other theoretical models could be used or created to assess digital health for individuals with different comfort levels with technology. For example, the Senior Technology Acceptance Model has been developed to assess older adults’ comfort with technology in Hong Kong [57].

### Limitations

There are opportunities to pursue a better understanding of medication adherence and to measure the impact on clinical practice. Currently, there is no consensus about methods to assess medication adherence, which makes it difficult to compare adherence rates across studies. The most frequently used method for assessing adherence is self-report, a subjective assessment of adherence; while cost-effective, self-report is often not as reliable as objective measurements, such as serum drug level or pill count [9]. Additionally, there were studies that addressed treatment adherence or self-management that did not meet the inclusion criteria for this integrative review because MA outcomes were not addressed as a study outcome or medication adherence outcome was not reported.

### Conclusions

This integrative review was conducted to examine the types of digital health technologies that have targeted medication adherence in the adult population, aged 18 years and older. Digital health included a number of technologies to foster medication adherence, including IVR, SMS text messaging, telehealth, and Web-based software. In some chronically ill populations, the digital health technology interventions that were reviewed fostered v via one-way communication to the patient or two-way communication between the patient and health care provider [43,48]. Two-way communication occurred through patient timely reporting of monitored results, such as blood glucose and BP to the health care provider to receive feedback about care [40]. Digital health technologies were found to be diverse and the populations studied varied in size, ethnicity, and age range. There remains ample opportunity to enhance patient and provider communication via digital technology as new mobile and electronic media emerge, especially in populations increasingly familiar with mobile phones, tablets, and other mobile communication devices.

### Relevance to Practice and Research

Nonadherence by adults is a significant public health problem and there are opportunities to better understand the role of digital health interventions for this population [3]. Digital health interventions provide cost-effective strategies as an adjunct to medication adherence management [43,49]. Future interventions should address the use of digital health interventions for medication adherence using evidence-based systematic frameworks to ensure this technology provides high-quality alternatives. This is a prominent area for future research considering the availability of technology among adults globally. Moreover, study findings suggest that digital health interventions can improve short-term medication adherence. Digital health interventions could help reduce health disparities related to nonadherence in chronically ill populations, such as those with diabetes and hypertension, where these interventions are used in combination with other treatments for those seeking to improve medication adherence. These modalities need further exploration among younger and much older populations and over longer durations to document sustainability of medication adherence.

## Appendix

Multimedia Appendix 1 Summary of studies included.

## Abbreviations

BP: blood pressure
CCM: Chronic Care Model
eCCM: eHealth-enhanced Chronic Care Model
eHealth: electronic health
IVR: interactive voice response
MA: medication adherence
MED: MEssaging for Diabetes
MeSH: Medical Subject Headings
mHealth: mobile health
MMAS: Morisky Medication Adherence Scale
PRISMA: Preferred Reporting Items for Systematic Reviews and Meta-Analyses
RCT: randomized controlled trial
SDSCA: Summary of Diabetes Self-Care Activities
SMASH: Smartphone Medication Adherence Stops Hypertension
SMS: short message service
TEACH-DM: Tailored Case Management for Diabetes and Hypertension
TExT-MED: Trial to Examine Text-based mHealth for Emergency department patients with Diabetes
