# Supplementary material for: Digital Health for Medication Adherence in Adult Diabetes or Hypertension: An Integrative Review
Source: JMIR Diabetes. 2017 Aug 16;2(2):e20. doi: 10.2196/diabetes.8030 (PMC6238839; doi:10.2196/diabetes.8030)
Supplement: Multimedia Appendix 1 [file diabetes_v2i2e20_app1.pdf]

Multimedia Appendix 1: Digital health technology and medication adherence
Summary of included studies.

Author, year (country)	Sample, intervention length, age & study purpose	Intervention	Medication adherence findings
Aikens et al 2014 (USA) [40]	N=301 with diabetes; 3-6 months; 66.7± 9.8; Characterize changes in diabetes self-management and psychological distress associated with a mobile health interactive voice response (IVR) self-management response program, Observational	Weekly IVR calls assessing health status and self-care and providing tailored pre-recorded self-management support messages; informal caregiver and patient clinician were notified when patients reported significant problems	Significant pre-post improvement in medication adherence using MMAS 8-item with significant improvement in patients' IVR-reported frequency of weekly medication adherence (MA).
Arora et al 2014 (USA) [41]	N=128 with diabetes; 6 months; 50.7 (SD 10.2); Determine whether scalable, low cost, unidirectional text message-based mobile health intervention (TExT-MED) improves clinical outcomes, increases health behaviors, and decreases Emergency Department utilization in a safety net population, RCT	TExT-MED group received 2 daily text messages in English or Spanish	Self-reported MA measured using the MMAS 8-item and improved from 4.5 to 5.4 in TExT-MED group compared with a net decrease of -0.1 in the controls.
Bobrow et al 2016 (South Africa) [42]	N=1372 with high blood pressure; 12 months; 53.9 – 54.3 (SD 11.5); Assessed effect of automated treatment adherence support delivered via mobile phone short message system (SMS) on blood pressure, RCT	SMS-Text Adherence Support (StAR); 3 arms – information only, interactive SMS text messaging, or usual care.	Self-reported MA and refill data: overall number of participants who had at least 80% of proportion of days of medication covered for blood pressure-lowering medication for the 12 month period<br>62.8% - information only group

			59.7% interactive group<br>49.4% usual care group
Davidson et al 2015 (USA) [43]	N=38; 3 month; 47.5 SD 11.8; Evaluate a smartphone MA Stops Hypertension (SMASH) program.<br><br>Hypothesized that participants in the SMASH condition would demonstrate significantly greater increased MA and decreased BP compared to participants in the treatment-as-usual condition, RCT	SMASH program patient-<br><br>centered, theory-<br><br>guided, iterative design process;<br><br>electronic medication trays<br><br>provided reminder signals<br><br>and SMS reminded subjects<br><br>to monitor BP with<br><br>Bluetooth-<br><br>Enabled monitors.<br><br>Motivational and reinforcement text messages sent to participants based on levels of adherence.	MA defined by the percent of SMS reminders over the past day(s). MA was 92 ± 0.09 for all participants in the SMASH group and .98 ± 0.03 for African American, and 0.86 ± 0.1 for Hispanics (0-1, 1 = fully compliant).
Edelman et al 2015 (USA) [44]	N=377; 24 months; 58.7 (SD 10.8); Evaluate a behavioral intervention among community patients with poorly controlled diabetes and comorbid hypertension, RCT	Tailored Case Management for Diabetes and Hypertension (TEACH-DM). All patients received call from nurse. Intervention group received tailored text diabetes and HTN focused content; Control group received non-tailored non-interactive information.	MA obtained by self-report and dichotomized into perfect verses imperfect. For the diabetes intervention, non-adherent (medication-taking scale) were 26.9% and control 31.5%. For the hypertension intervention, non-non-adherent (medication-taking scale) were 43.0% and control 42.9%.

Katalenich et al 2015 (USA) [45]	N= 98; 6 months; 59 years (mean reported); Evaluate utility and cost-effectiveness of automated DRMS in glycemic control, RCT	Diabetes Remote Monitoring and Management System (DRMS) in glycemic control verses usual care. Text messages or phone calls to remind patients to report results via automated system. No interaction unless severely high or low glucose.	MA measured using MMAS 8-item. None of the participants scored in the high medication adherence category. Intervention group had higher adherence than control group at each measurement. Overall improvements in MA self-report were not significant.
Kim et al 2006 (South Korea) [46]	N=45 (33 completers); 12 weeks; 43.5 ±12.6; Investigate effect of nurse SMS by cellular phone and internet on A1C and adherence to diabetes control recommendations, RCT sub-study	SMS; intervention consisted of continuous education and reinforcement of diet, exercise, medication adjustment, and frequent self-monitoring of blood glucose levels.	Self-reported MA measured by diabetes self-care activities measure 4-item. Diabetes medication taking adherence increased 1.1 days per week at post-test compared to pre-test.
Migneault et al 2012 (USA) [48]	N=337; 8 months; 56.6 (SD 11.0); Evaluate a culturally adapted, automated telephone system to help hypertension, RCT	2 arm - Automated, multi-behavior intervention or education-only control.	Medication adherence measured using a 7-item version of MMAS. Baseline mean MA low in both groups and although the treatment group's adjusted MMAS scores improved by 0.19 points relative to controls, this change was not statistically significant.
Nelson et al 2016 (USA) [47]	N=80; 3 months; 50.1 ±10.5; Tested efficacy of SMS text messaging service and IVR intervention to promote adherence among adults with T2D, Quasi-experimental design	MEssaging for Diabetes (MED) SMS/IVR intervention using SuperEgo communication platform to deliver and tailor text messages and voice	Barriers to MA assessed using items from the Diabetes Medication Knowledge Questionnaire, Medicines for Diabetes Questionnaire and Barriers to Diabetes

		communications to promote MA	Adherence measure and Medication Adherence Self-Efficacy Scale. Medication Adherence assessed using the SDSCA-MS. There was no difference in adherence between the intervention group and control group at 3 months. MED had a positive, short-term impact on adherence.
Nundy et al 2014 (USA) [49]	N=74; 54.1 ± 9.3 years; 6 months; Investigate the behavioral effects of a theory-driven, mobile phone-based intervention that combines automated text messaging and remote nursing, using an automated, interactive text messaging system, Mixed methods observational cohort study	CareSmarts was a theoretically guided, mobile phone-based intervention for diabetes behavior support delivered through a web-based software. Patients received educational messages and reminders and texted back responses to questions.	Two self-reported measures: Diabetes Self-Care Activities (DSCA) measure of weekly adherence and MMAS 4-item. At both 3 months ($P < .01$) and 6 months ($P = .02$), MMAS improved compared to baseline; however, no change in weekly MA was observed.
Shane-McWhorter et al 2014 (USA) [22]	N=125; 50.6 (mean); 6 months; Evaluate the use of a telemonitoring system using IVR to improve diabetes and hypertension outcomes, Non-randomized prospective observational pre-intervention-post-intervention study	Telemonitoring with asynchronous measurements transmitted from the patient to a remote care coordinator pharmacist certified diabetes educator.	MA measured using an 8-item validated questionnaire. MA for diabetes and hypertension improved but was not significant (P=0.09 and P=0.054 respectively)
Wakefield et al 2011 (USA) [26]	N= 304; 6 months; 68.0 (SD 9.8); Evaluate the efficacy of a nurse-	Closed surveillance via home telehealth device and nurse care	MA measured using the Self-Reported Medication Taking scale

	managed home telehealth intervention to improve outcomes in veterans with comorbid diabetes and hypertension, RCT	management; 3 arms: high intensity group – algorithm programed into device; low-intensity group – small subset of questions with branching algorithm; & usual care.	and a validated regimen adherence scale. MA improved over time for all groups, but there were no differences among the three groups.
Wild et al 2016 (UK) [50]	N=321; 9 months; 61 (SD 9.8); Investigated whether health professional review of telemetrically transmitted self-monitored glucose results in improved glycemic control in people with poorly controlled T2D, RCT	Supported telemonitoring intervention involved self-measurement and transmission to a secure website of twice-weekly morning and evening glucose for review by family practice clinicians; control group received usual care.	MA measured using MMAS; No significant difference identified between groups in adherence to medication.
